# Identification of key proteins in the signaling crossroads between wound healing and cancer hallmark phenotypes

**DOI:** 10.1038/s41598-021-96750-5

**Published:** 2021-08-26

**Authors:** Andrés López-Cortés, Estefanía Abarca, Leonardo Silva, Erick Velastegui, Ariana León-Sosa, Germania Karolys, Francisco Cabrera, Andrés Caicedo

**Affiliations:** 1 Facultad de Ciencias de la Salud Eugenio Espejo, Universidad UTE, Quito, Ecuador; 2 Latin American Network for the Implementation and Validation of Clinical Pharmacogenomics Guidelines (RELIVAF-CYTED), Madrid, Spain; 3 RNASA-IMEDIR, Computer Science Faculty, Universidad of A Coruna, A Coruña, Spain; 4 Carrera de Biotecnología, Universidad Politécnica Salesiana UPS, Quito, Ecuador; 5 Instituto de Investigaciones en Biomedicina iBioMed, Universidad San Francisco de Quito USFQ, Quito, Ecuador; 6 Grupo de Investigación y Desarrollo en Ciencias Aplicadas a los Recursos Biológicos, Universidad Politécnica Salesiana, Quito, Ecuador; 7 Colegio de Ciencias de la Salud, Escuela de Medicina Veterinaria, Universidad San Francisco de Quito USFQ, Quito, Ecuador; 8 Colegio de Ciencias de la Salud, Escuela de Medicina, Universidad San Francisco de Quito USFQ, Quito, Ecuador; 9 Mito-Act Research Consortium, Quito, Ecuador; 10 Sistemas Médicos SIME, Universidad San Francisco de Quito USFQ, Quito, Ecuador

**Keywords:** Cancer, Molecular medicine, Oncology

## Abstract

Wound healing (WH) and cancer seem to share common cellular and molecular processes that could work in a tight balance to maintain tissue homeostasis or, when unregulated, drive tumor progression. The “Cancer Hallmarks” comprise crucial biological properties that mediate the advancement of the disease and affect patient prognosis. These hallmarks have been proposed to overlap with essential features of the WH process. However, common hallmarks and proteins actively participating in both processes have yet to be described. In this work we identify 21 WH proteins strongly linked with solid tumors by integrated TCGA Pan-Cancer and multi-omics analyses. These proteins were associated with eight of the ten described cancer hallmarks, especially avoiding immune destruction. These results show that WH and cancer's common proteins are involved in the microenvironment modification of solid tissues and immune system regulation. This set of proteins, between WH and cancer, could represent key targets for developing therapies.

## Introduction

All eukaryotic cells share similar growth, proliferation, migration, and survival pathways. However, their mechanisms of control and differentiation are diverse and could be deregulated giving rise to disease. Mutations can accumulate in normal cells throughout a person’s lifetime, some of which may be silent, while others can alter key cellular functions and lead to cancer^1^. Cancer cells differ from normal cells as they reproduce without control, wanting to prevail and survive through specific cancer hallmarks, such as resisting cell death and sustaining proliferative signaling that provides them with a selective advantage over normal cells^2,3^. Interestingly, cells in wounded areas proliferate for tissue repair and survival under the control of the organism in ways very similar to cancer hallmarks^4^. Identifying the overlapping survival and proliferative mechanisms between normal cells in wounds and cancer could be crucial for the development of new therapies that may lead to the prevention and inhibition of cancer progression.

The idea of molecular similarities between tumors and wounds has been in the literature for more than 150 years^5^. Back in the 1970s, Haddow raised the question of tumor production as a way of ‘overhealing’^6^. Similarly, Harold Dvorak published a work in 1986 entitled ‘Tumors: Wounds that do not heal’^7^. These contributed to the current concept that wound repair and cancer share cellular and molecular processes that are controlled in normal wound healing (e.g. self-limited process) but dysregulated in cancer (e.g. continuous activation of the pathways involved)^4,8^. Key and overlapping proteins between wound healing (WH) and cancer could be identified in both of these processes leading to the recognition of essential master proteins to stop cancer progression.

WH is a complex and evolved defense mechanism that integrates a cascade of cellular responses in the site of injury to restore tissue homeostasis, epidermal integrity, and the skin barrier function^9–11^. This process consists of four highly programmed and discrete yet overlapping phases: hemostasis, inflammation, proliferation, and remodeling^12,13^. These phases occur in a continuous and regulated manner. Hence, lack of control or lengthening of these processes driven by factors such as aberrancies in gene expression can lead to a delayed wound repair or non-healing wounds^14^. Such delayed processes, especially in the inflammatory response of chronic wounds, have been widely compared to the inflammation in cancer^5,6^.

Hanahan and Weinbergs’ hallmarks of cancer highlight the key biological processes underlying the development, growth, and progression of tumors. Some of these cancer enabling characteristics coincide with similar mechanisms during the WH process, particularly the inflammatory and proliferative response. Furthermore, it has recently been proposed that the hallmarks of cancer are also the hallmarks of WH. However, not all cancer hallmarks have parallels in WH. Such is the case for enabling replicative immortality, genomic instability and mutation occurrence^4^.

Nowadays, the scientific community still lacks information and has not reached a consensus on the set of specific genes and proteins that both activate the mechanisms of WH and are involved with cancer. The aim of the present work is to identify for the first time which WH proteins are enriched in the cancer hallmark phenotypes by using an integrated TCGA Pan-Cancer and multi-omics analyses in order to reveal novel therapeutic targets for anti-cancer therapy.

## Methods

### Protein sets

In order to identify WH proteins significantly enrolled in cancer, we analyzed two protein sets. On one hand, we retrieved 347 human proteins related to the “wound healing” term from the Gene Ontology (GO) database (GO:0042060) (http://www.geneontology.org)^15,16^, and the David Bioinformatics Resource (https://david.ncifcrf.gov/)^17^. On the other hand, to identify which WH proteins were already catalogued as cancer drivers, we retrieved 874 cancer driver proteins from the intOGen framework (https://www.intogen.org). This analysis helps to identify the mechanism of action of proteins across tumor types^18^. Then, we used The Catalogue of Somatic Mutations in Cancer (COSMIC) Cancer Gene Census (CGC) (https://cancer.sanger.ac.uk/census), that is an expert-curated description of the proteins driving cancer used in oncology research^19^. Both protein sets, the WH and the cancer, are provided in detail in the Supplementary Tables 1 and 2, respectively.

### OncoPrint of genomic, transcriptomic and proteomic alterations according to the TCGA Pan-Cancer Atlas

After identifying the set of WH-related proteins, we retrieved their genomic, transcriptomic and proteomic alterations in the Pan-Cancer Atlas (PCA) project which belongs to The Cancer Genome Atlas (TCGA) consortium^20,21^. The TCGA Pan-Cancer types were adrenocortical carcinoma (ACC), bladder urothelial carcinoma (BLCA), brain lower grade glioma (LGG), breast invasive carcinoma (BRCA), cervical squamous cell carcinoma and endocervical carcinoma (CESC), cholangiocarcinoma (CHOL), colon adenocarcinoma (COAD), esophageal carcinoma (ESCA), glioblastoma multiforme (GBM), head and neck squamous cell carcinoma (HNSC), kidney renal clear cell carcinoma (KIRC), kidney chromophobe (KICH), liver hepatocellular carcinoma (LIHC), lung adenocarcinoma (LUAD), lung squamous cell carcinoma (LUSC), mesothelioma (MESO), ovarian serous cystadenocarcinoma (OV), pancreatic adenocarcinoma (PAAD), prostate adenocarcinoma (PRAD), sarcoma (SARC), skin cutaneous melanoma (SKCM), stomach adenocarcinoma (STAD), testicular germ cell tumors (TGCT), thymoma (THYM), thyroid carcinoma (THCA), uterine carcinosarcoma (UCS), uterine corpus endometrial carcinoma (UCEC), and uveal melanoma (UVM).

According to the Genomics Data Commons of the National Cancer Institute (https://portal.gdc.cancer.gov/), and the cBioPortal database (http://www.cbioportal.org/)^22,23^, the copy number variant (CNV) amplifications and CNV deep deletions were identified using GISTIC2.0, a computational approach that facilitates sensitive and confident localization of CNV in human cancers^24^; the inframe, truncating and missense driver mutations were identified through whole exome sequencing; the mRNA high and mRNA low alterations were analyzed through RNA sequencing V2 RSEM where the expression Z-scores of tumor samples were compared to the expression distribution of all log-transformed mRNA expression of adjacent normal samples in each cohort^25^; and the high and low protein expressions were measured by reverse-phase protein array (RPPA)^26^. Subsequently, we analyzed these genomic, transcriptomic and proteomic alterations belonging to WH genes/proteins of 10,711 individuals with 28 different cancer types.

To generate the OncoPrint encompassing the most significantly altered WH genes/proteins we: (1) calculated the number of alterations per gene and per TCGA Pan-Cancer type; (2) normalized the frequency of alterations dividing the number of alterations per gene by the number of individuals per each cancer cohort; (3) calculated the mean frequency per gene and per alteration type considering all Pan-Cancer types; (4) identified the most altered WH genes/proteins taking into account as a cutoff the mean frequency of all genes/proteins; and (5) validated the most significantly altered WH genes/proteins comparing the alteration frequencies between the group of genes/proteins with the highest alteration frequencies (cutoff > mean frequency) versus the group of genes/proteins with the lowest alteration frequencies (cutoff < mean frequency) by using the Mann–Whitney U test (*P* < 0.001). Lastly, we applied the Bonferroni correction test (*P* < 0.001) to perform a multiple comparison between 6) the whole TCGA Pan-Cancer alterations, and 7) the TCGA Pan-Cancer types.

### Patient-derived xenografts

With the aim of generating a deeper understanding about the underexpression and overexpression of WH genes in a given tissue, we analyze their behavior in vivo by using bioinformatic resources. The Jackson Laboratory PDX resource (http://tumor.informatics.jax.org/%20mtbwi/pdxSearch.do) comprises 455 PDX models originating from 34 different primary sites^27^. The PDX models were genomically characterized to identify copy number variants, somatic mutations, and transcriptional profiles. Here, we analyzed the expression levels of the 347 WH genes taking into account Z-scores ≥ 2 as overexpressed genes and Z-scores ≤ − 2 as underexpressed genes in the PDX lineages. The gene expression is displayed as a Z-score which measures each gene’s model-specific expression in comparison with that gene in all models assayed by the same platform. Additionally, we have calculated a two-tailed *P*-value per each gene Z-score and have visualized the Z-score distribution of the transcriptional profile in a plot. Lastly, all overexpressed (Z-score ≥ 2) and underexpressed genes (Z-score ≤  − 2) with significant *P*-values (*P* < 0.05) encompassed the WH genes from the patient-derived xenograft approach.

### Wound healing protein–protein interactome network

In order to identify the most essential protein interactions, a WH protein–protein interactome (WH-PPi) network was created by using the human proteome of the Cytoscape StringApp, taking into account zero node addition and the highest confidence interactions (cutoff = 0.9) related to experiments, databases, and co-expression^28,29^. The degree of centrality represents the number of edges the nodes have in a network^30–32^, and this centrality index was calculated using the CytoNCA app^33^. Nodes and edges were organized through the organic layout, and the WH-PPi network was visualized through the Cytoscape software v.3.7.1^34^. Lastly, the interactome network analysis considered all WH proteins with at least one high-confidence interaction (cutoff = 0.9) in the human proteome.

Regarding the cancer driver proteins encompassing the WH-PPi network, we compared the degree centrality between the cancer driver nodes and the wound healing nodes by using the Mann–Whitney U test (*P* < 0.001) in order to determine a correlation between both groups of proteins.

### Shortest paths from wound healing proteins to cancer hallmark phenotypes

CancerGenNet (https://signor.uniroma2.it/CancerGeneNet/) is a resource that links proteins that are frequently altered in all cancer types to cancer hallmark phenotypes^35^. This bioinformatic tool, curated by SIGNOR^36^, is based on experimental information that allows to infer likely paths of causal interactions linking proteins to cancer phenotypes. According to Iannuccelli et al.^35^, the shortest paths from proteins to cancer phenotypes were programmatically implemented using the shortest path function of *igraph* R package, obtaining a distance score. Hence, we analyzed the distance score of shortest paths from wound healing proteins to cancer hallmark phenotypes. Positive regulations were calculated for tumor-promoting inflammation, inducing angiogenesis, cell differentiation, reprogramming of energy metabolism, activating invasion and metastasis, and sustaining proliferative signaling to better understand the signaling crosstalk between wound healing proteins and cancer hallmarks, and negative regulations were calculated for resisting cell death. Additionally, we performed the multiple comparison test as Bonferroni correction (*P* < 0.001) to compare the mean of the distance score of shortest paths across seven cancer hallmark phenotypes.

### Functional enrichment analysis

The functional enrichment analysis gives curated signatures of protein sets generated from omics-scale experiments^37^. Therefore, we performed the functional enrichment analysis of the most altered WH proteins identified through multi-omics approaches (PCA, PDXs, WH-PPi network, and shortest paths to cancer phenotypes). The enrichment was analyzed using g:Profiler version e101_eg48_p14_baf17f0 (https://biit.cs.ut.ee/gprofiler/gost) to obtain significant annotations (Benjamini–Hochberg FDR *q* < 0.001) related GO biological processes, the Kyoto Encyclopedia of Genes and Genomes (KEGG) signaling pathways, and Reactome signaling pathways^38–40^.

### Statistical analyses

We performed a multiple comparison test using the Bonferroni correction (significant level of *P* < 0.001 and a 95% confidence interval) to analyze: (1) significant differences of genomic, transcriptomic and proteomic alteration frequencies across alteration types (mRNA high, mRNA low, CNV amplification, CNV deep deletion, protein high, protein low, driver mutation, and fusion gene), and (2) significant differences of genomic, transcriptomic and proteomic alteration frequencies across 28 TCGA Pan-Cancer types. We also validated the most significant WH genes/proteins that encompassed the OncoPrint comparing the alteration frequencies above and below the cutoff by using the Mann–Whitney U test (*P* < 0.001). The significance of gene expression in patient-derived xenografts was considered by using Z-scores and *P*-values. Therefore, genes with Z-score ≥ 2 and two-tailed *P* < 0.05 mean significant overexpression, and genes with Z-score ≤ − 2 and two-tailed *P* < 0.05 mean significant underexpression. The WH-PPi network takes into account the highest confidence interactions (cutoff = 0.9). We validated the nodes encompassing the WH-PPi network comparing the degree centrality of them with the cancer driver proteins by using the Mann–Whitney U test. Additionally, we calculated the mean of the distance score of the shortest paths across cancer hallmark phenotypes by using the Bonferroni correction as a multiple comparison test (*P* < 0.001). Lastly, the functional enrichment analysis of the key proteins in the signaling crossroad between cancer hallmarks and wound healing was performed using g:Profiler that determines the most significant GO: biological processes, KEGG signaling pathways, and Reactome signaling pathways with Benjamini–Hochberg FDR *q* < 0.001.

## Results

### OncoPrint of genomic and proteomic alterations according to the TCGA Pan-Cancer Atlas

**Figure 1:**
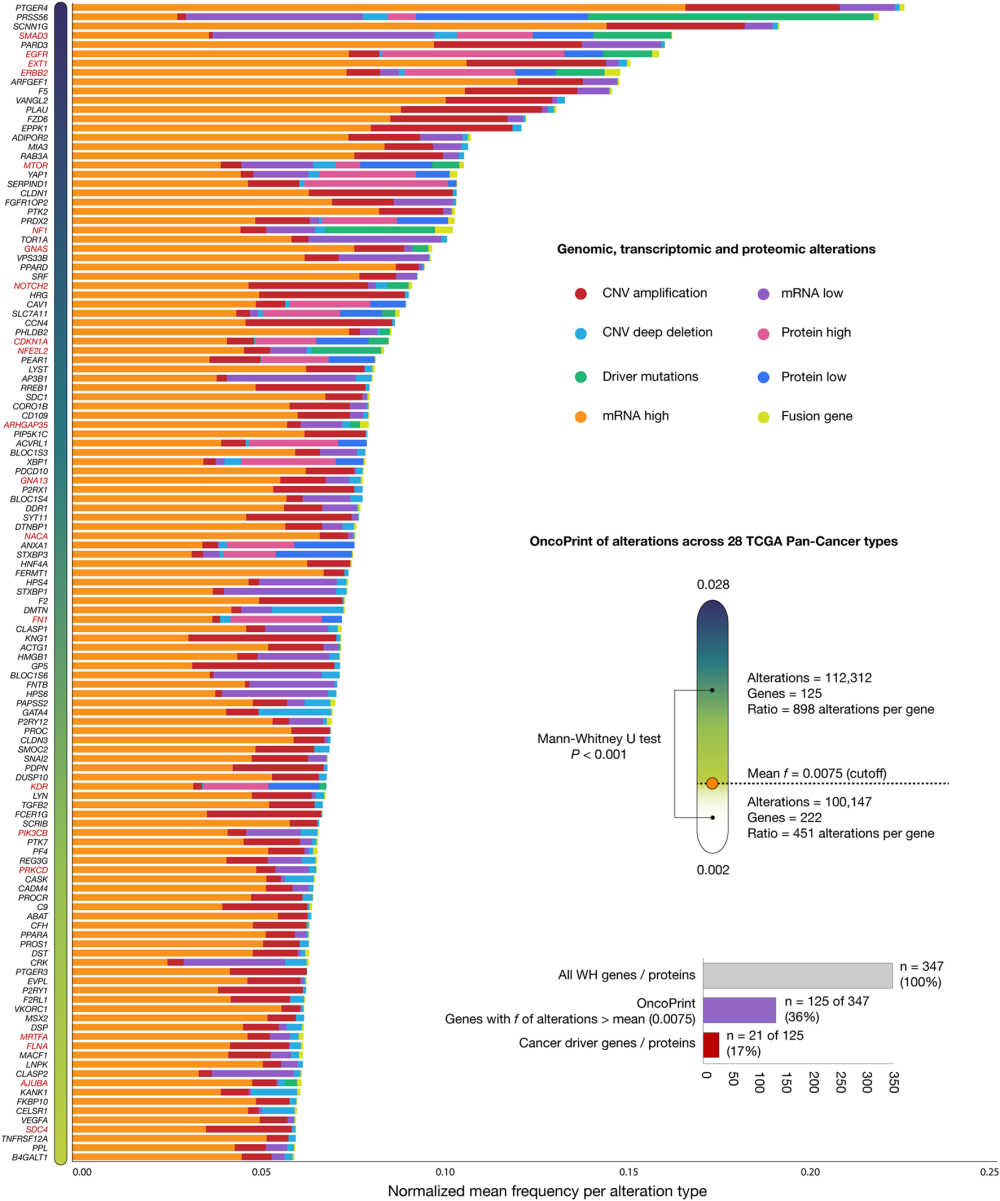
OncoPrint of genomic, transcriptomic and proteomic alterations across 28 TCGA Pan-Cancer types. Ranking of the most altered wound healing genes/proteins (n = 125) taking into account the mean frequency of alterations (cutoff = 0.0075). Lastly, the list includes 21 cancer driver proteins. Lastly, the OncoPrint was performed by using data from the cBioPortal platform (https://www.cbioportal.org/)^22,23^.

We have identified 212,459 genomic, transcriptomic and proteomic alterations in the 347 WH genes/proteins belonging to 10,711 individuals with 28 different TCGA Pan-Cancer types. Figure 1 and Supplementary Tables 3, 4, 5, 6, 7, 8, 9, 10 details the OncoPrint of alterations (CNV amplification, CNV deep deletion, mRNA high, mRNA low, protein high, protein low, driver mutation, and fusion gene) involving 1084 (10.1%) individuals with BRCA, 594 (5.5%) with COAD, 585 (5.5%) with GBM, 585 (5.5%) with OV, 566 (5.3%) with LUAD, 529 (4.9%) with UCS, 529 (4.9%) with UCEC, 523 (4.9%) with HNSC, 514 (4.8%) with LGG, 512 (4.8%) with KIRC, 499 (4.7%) with THCA, 494 (4.6%) with PRAD, 487 (4.5%) with LUSC, 442 (4.1%) with SKCM, 440 (4.1%) with STAD, 411 (3.8%) with BLCA, 372 (3.5%) with LIHC, 297 (2.8%) with CESC, 255 (2.4%) with SARC, 184 (1.7%) with PAAD, 182 (1.7%) with ESCA, 149 (1.4%) with TGCT, 123 (1.1%) with THYM, 87 (0.8%) with ACC, 87 (0.8%) with MESO, 80 (0.7%) with UVM, 65 (0.6%) with KICH, and 36 (0.3%) with CHOL. After normalizing the frequency of alterations which consisted of dividing the number of alterations per gene by the number of individuals per each cancer cohort, the overall analysis revealed 125 (36%) significantly altered WH genes/proteins (Mann–Whitney U test *P* < 0.001) with alteration frequencies higher than the average (cutoff > 0.0075). Lastly, the top ten WH genes/proteins with the highest alteration frequencies were *PTGER4*, *PRSS56*, *SCNN1G*, *SMAD3*, *PARD3*, *EGFR*, *EXT1*, *ERBB2*, *ARFGEF1*, and *F5*.

**Figure 2:**
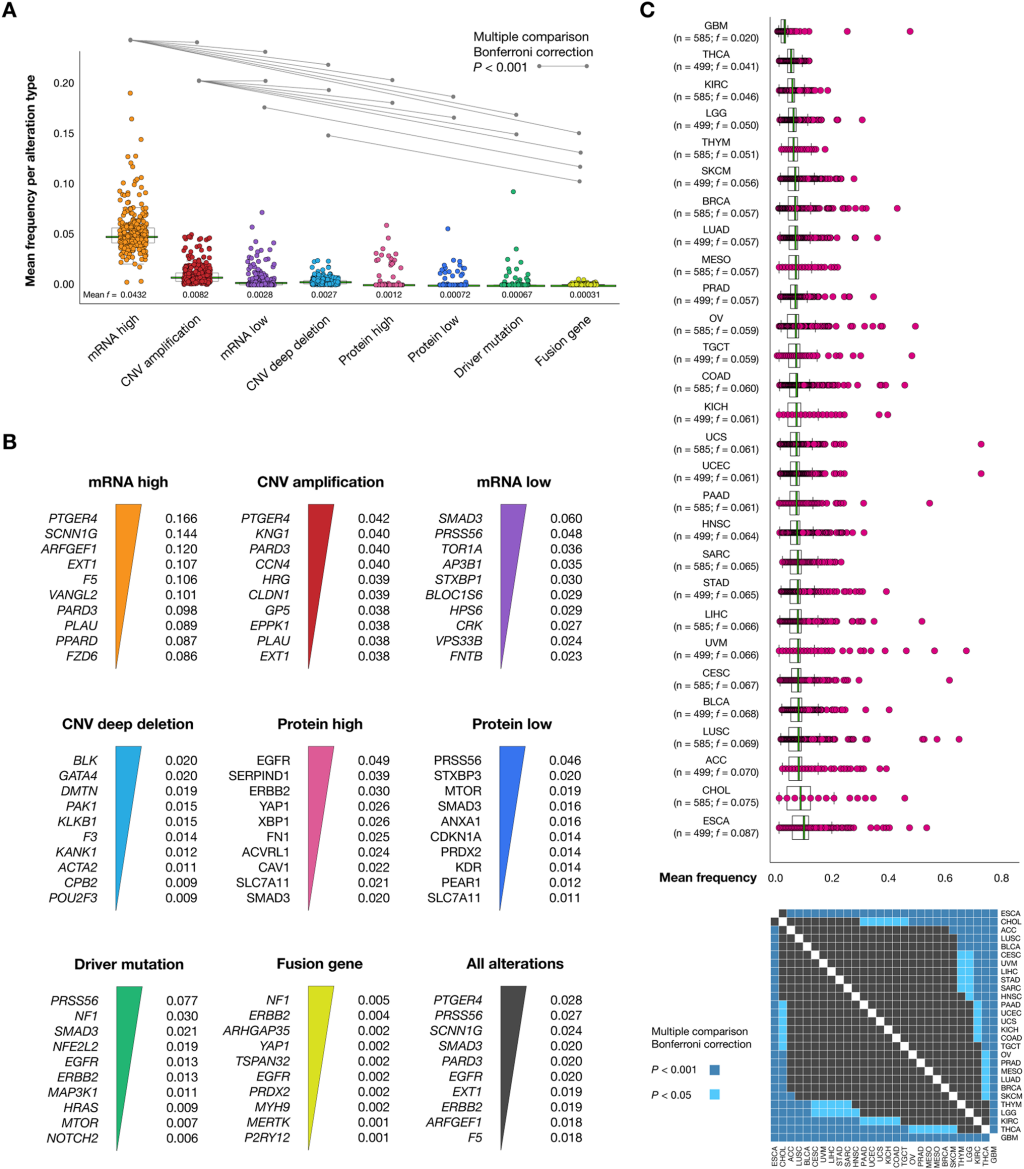
Frequency of genomic, transcriptomic and proteomic alterations per TCGA Pan-Cancer type. (**A**) Mean frequency per alteration type and significant Bonferroni correction (*P* < 0.001) of mRNA high, CNV amplification, mRNA low and CNV deep deletion in comparison with other alterations. (**B**) Ranking of the most altered genes/proteins per alteration type. (**C**) Ranking of the most altered TCGA Pan-Cancer types according to the mean frequency of alterations. Lastly, a pairwise map of significant Bonferroni correction (*P* < 0.001) across TCGA Pan-Cancer types.

The most common alteration type with a *f* mean of 0.0432 was mRNA high, followed by CNV amplification (0.0082), mRNA low (0.0028), CNV deep deletion (0.0027), protein high (0.0012), protein low (0.0007), driver mutations (0.0007), and fusion gene (0.0003). We performed the Bonferroni correction as a multiple comparison test to obtain significant alterations (*P* < 0.001) through the TCGA Pan-Cancer types. Therefore, we detected that mRNA high and CNV amplification were significantly altered (*P* < 0.001) across all genomic, transcriptomic, and proteomic alterations (Fig. 2A and Supplementary Table 11). Additionally, genes/proteins with the highest alteration frequencies were *PTGER4*, *SCNN1G*, and *ARFGEF1* with mRNA high alterations; *PTGER4*, *KNG1*, and *PRAD3* with CNV amplifications; *SMAD3*, *PRSS56*, and *TOR1A* with mRNA low alterations; *BLK*, *GATA4*, and *DMTN* with CNV deep deletions; EGFR, SERPIND1, and ERBB2 with protein high alterations; PRSS56, STXBP3, and MTOR with protein low alterations; *PRSS56*, *NF1*, and *SMAD3* with truncating, inframe and missense driver mutations; and, *NF1*, *ERBB2*, and *ARHGAP35* with fusion genes. The complete information of alteration frequencies per gene has been detailed in Fig. 2B and Supplementary Table 11.

Figure 2C shows the TCGA Pan-Cancer types with the highest means of alteration frequencies into WH genes/proteins. ESCA was the cancer type with the highest alteration frequency mean (*f* = 0.087), followed by CHOL (0.075), ACC (0.070), LUSC (0.069), BLCA (0.068), CESC (0.067), UVM (0.066), LIHC (0.066), STAD (0.065), SARC (0.065), HNSC (0.064), PAAD (0.061), UCEC (0.061), UCS (0.061), KICH (0.061), COAD (0.060), TGCT (0.059), OV (0.059), PRAD (0.057), MESO (0.057), LUAD (0.057), BRCA (0.057), SKCM (0.056), THYM (0.051), LGG (0.050), KIRC (0.046), THCA (0.041), and GBM (0.020). Additionally, we performed the Bonferroni correction as a multiple comparison test to obtain the most significantly altered TCGA Pan-Cancer types (*P* < 0.001). For instance, ESCA was the most significantly altered (*P* < 0.001), and GBM was the less significantly altered (*P* < 0.001) TCGA Pan-Cancer type. Lastly, the complete information of Bonferroni correction results across TCGA Pan-Cancer types is detailed in Fig. 2C and Supplementary Table 12.

### Patient-derived xenografts

**Figure 3:**
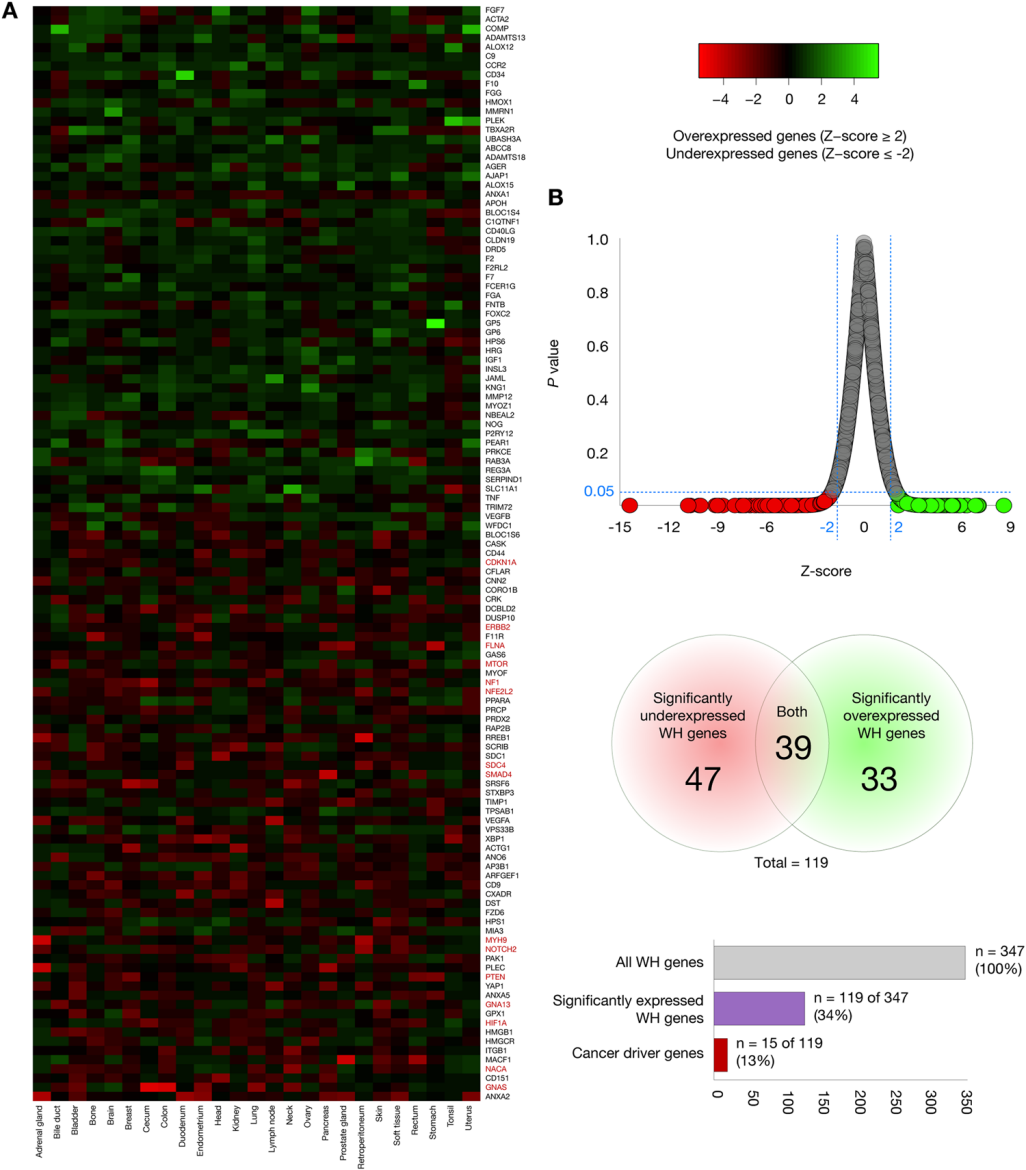
Patient-derived xenografts. (**A**) Heatmap of significantly overexpressed (Z-score ≥ 2) and underexpressed (Z-score ≤ 2) genes across 25 primary sites of tumor. (**B**) Plot of distribution of the transcriptional profile of WH genes considering their Z-scores and *P*-values per each cancer type. Lastly, the heatmap includes 15 cancer driver genes.

PDXs are in vivo models of human cancer types engrafted in mouse hosts for translational cancer research and therapy selection for individual patients^27^. We analyzed the gene expression levels of 347 proteins related to the wound healing term (GO:0042060). Figure 3A shows a heatmap of transcriptional expression where 119 WH genes were overexpressed (Z-score ≥ 2) and/or underexpressed (Z-score ≤ − 2) in 25 cancer types. Figure 3B shows a plot of distribution of the transcriptional profile of the 119 WH genes considering their Z-scores and two-tailed *P*-values. Of them, 33 were significantly overexpressed WH genes (*P* < 0.05), 47 were significantly underexpressed WH genes (*P* < 0.05), and 39 were both significantly overexpressed and underexpressed WH genes (*P* < 0.05). Regarding cancer drivers, we identified to *CDKN1A*, *ERBB2*, *FLNA*, *MTOR*, *NF1*, *NFE2L2*, *SDC4*, *SMAD4*, *MYH9*, *NOTCH2*, *PTEN*, *GNA13*, *HIF1A*, *NACA*, and *GNAS* as significantly expressed genes. Lastly, the overall analysis revealed 119 (34%) WH genes with significant expression in PDXs as detailed in the Supplementary Tables 13, 14, 15.

### Wound healing protein–protein interactome network

**Figure 4:**
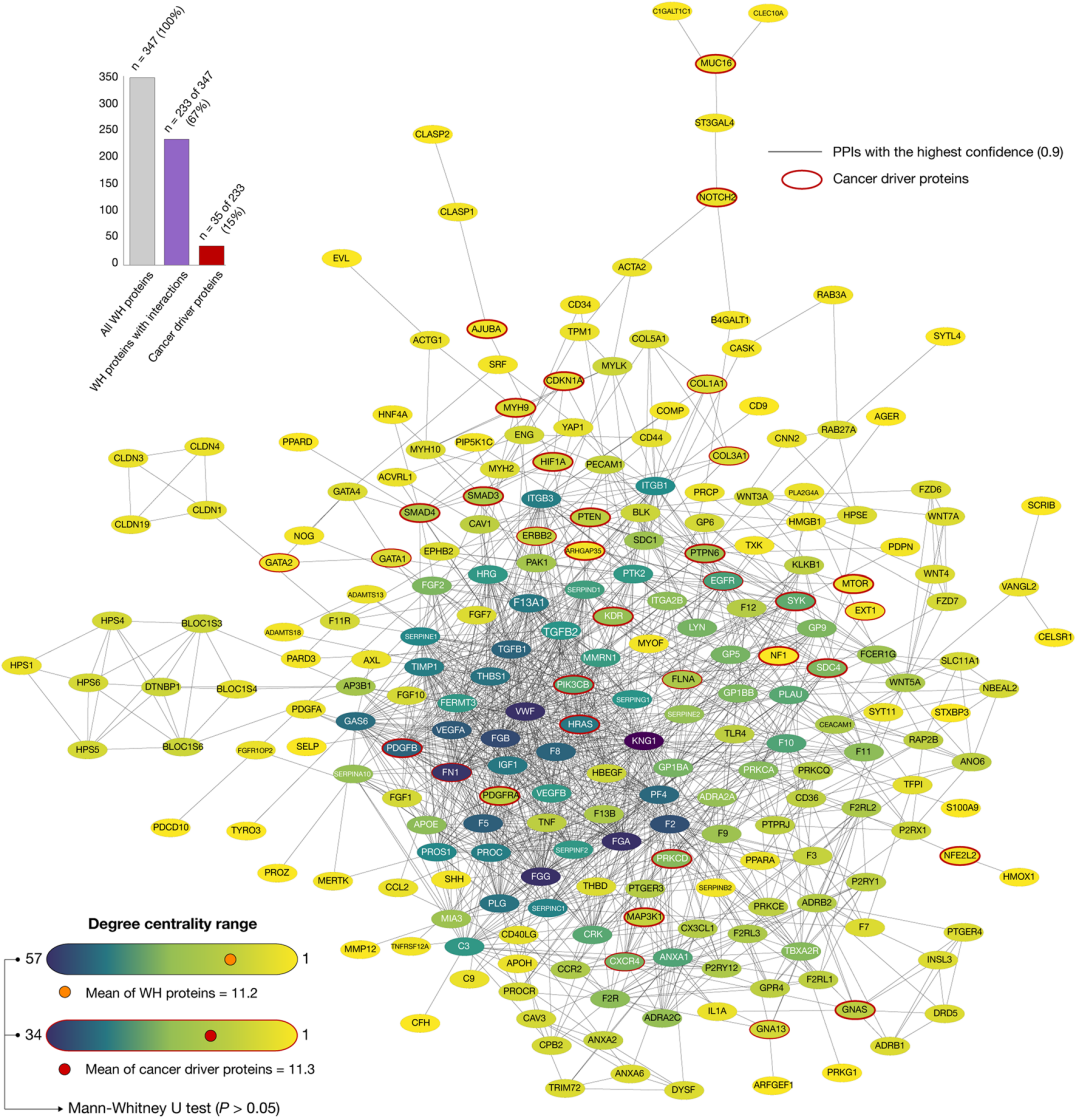
Wound healing protein–protein interactome network. Network of 233 wound healing proteins with at least one high confidence interaction (cutoff = 0.9). Darkest nodes represent proteins with the highest degrees of centrality (mean = 11.2). The WH-PPi network includes 35 cancer driver proteins with a degree centrality mean of 11.3. The Mann–Whitney U test showed a correlation of degrees of centrality between WH nodes and cancer driver nodes (*P* > 0.05). Lastly, the WH-PPi network was visualized through the Cytoscape software v.3.7.1^34^.

Figure 4 shows the WH-PPi network with a degree centrality mean of 11.2. KNG1, VWF, FGG, FGA, FN1, FGB, F2, VEGFA, F5, and TGFB1 were the top ten WH proteins with the highest degree of centrality. Figure 4 also shows the cancer driver proteins with a degree of centrality mean of 11.3. FN1, PDGFB, HRAS, EGFR, PIK3CB, SYK, CXCR4, PRKCD, KDR, and PTPN6 were the top ten cancer driver proteins with the highest degree of centrality. The comparison of degree centralities between the cancer driver nodes and the wound healing nodes showed a correlation between both networks with a not significant Mann–Whitney U test (*P* > 0.05). Lastly, the overall analysis revealed 233 (67%) WH proteins with at least one high-confidence interaction (cutoff = 0.9) in the human proteome as detailed in the Supplementary Table 16.

### Shortest paths from wound healing proteins to cancer hallmark phenotypes

**Figure 5:**
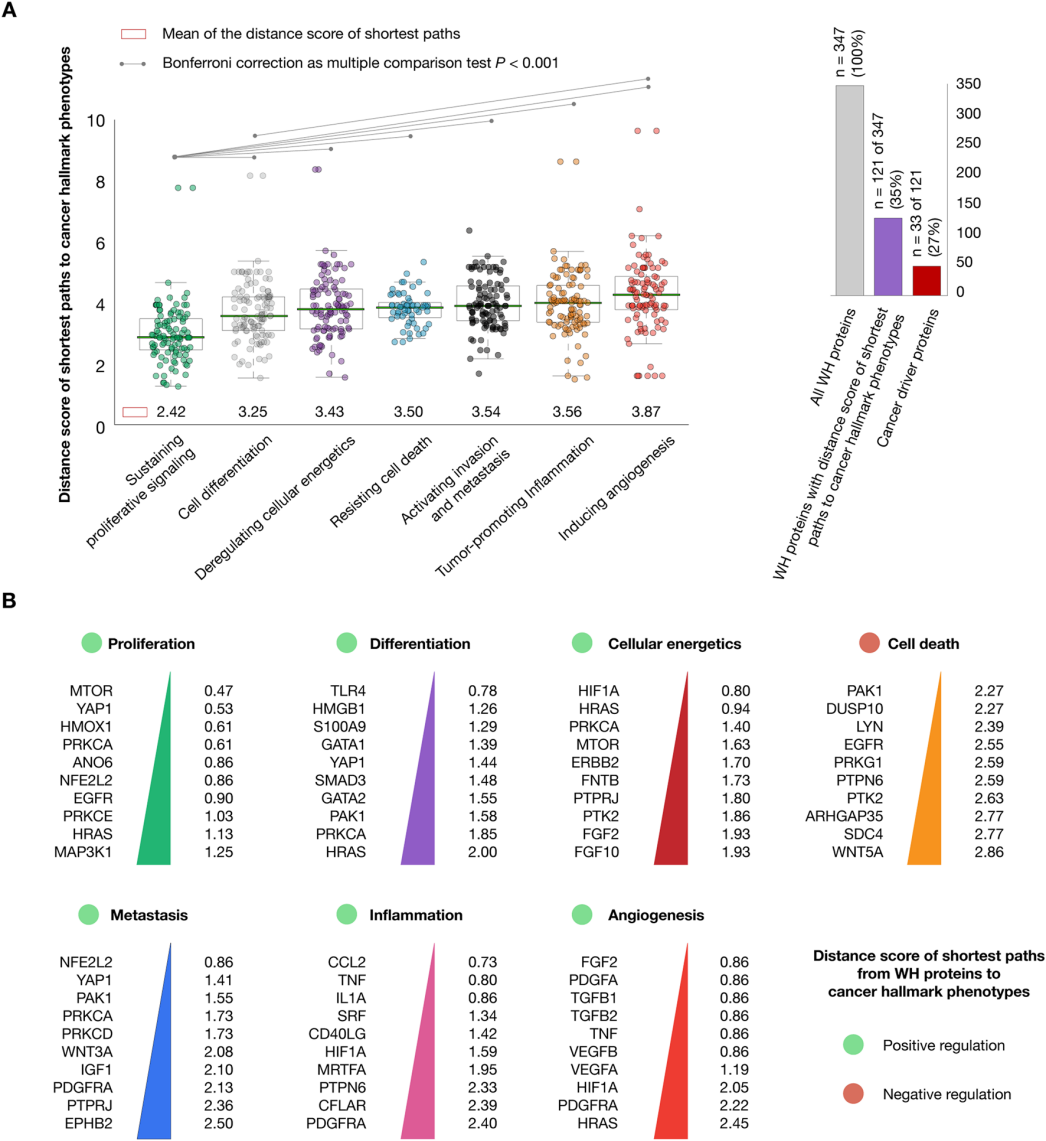
Shortest paths to cancer hallmark phenotypes. (**A**) Mean of the distance scores of shortest paths from wound healing proteins to cancer hallmark phenotypes and Bonferroni correction as multiple comparison test (*P* < 0.001). (**B**) Top ten wound healing proteins with the shortest distance scores of positive regulation to proliferation, differentiation, cellular energetics, metastasis, inflammation and angiogenesis, and negative regulation to resisting cell death. Lastly, the shortest paths to cancer hallmark phenotypes were analyzed by using data from CancerGenNet (https://signor.uniroma2.it/CancerGeneNet/)^35^.

We analyzed the 347 WH proteins by using CancerGeneNet software to find the distance score of the shortest paths to cancer hallmark phenotypes according to Iannuccelli et al.^35^. On the one hand, Fig. 5A revealed that the WH proteins had the shortest paths to proliferation (2.42), followed by differentiation (3.25), deregulating cellular energetics (3.43), resisting cell death (3.50), metastasis (3.54), tumor-promoting inflammation (3.56), and angiogenesis (3.87). The Bonferroni correction test showed that the WH proteins had significantly shorter paths to proliferation phenotype (*P* < 0.001) in comparison to the other cancer hallmarks. On the other hand, Fig. 5B shows the top ten WH proteins with the shortest paths to cancer hallmarks. MTOR had the shortest path to sustaining proliferative signaling (0.47), TLR4 to differentiation (0.78), HIF1A to deregulating cellular energetics (0.80), PAK1 to resisting cell death (2.27), NFE2L2 to metastasis (0.86), CCL2 to tumor-promoting inflammation (0.73) , and FGF2 to angiogenesis (0.86). Lastly, the overall analysis revealed 121 (35%) WH proteins with shortest paths to cancer hallmark phenotypes as detailed in Supplementary Table 17.

### Functional enrichment analysis

**Figure 6:**
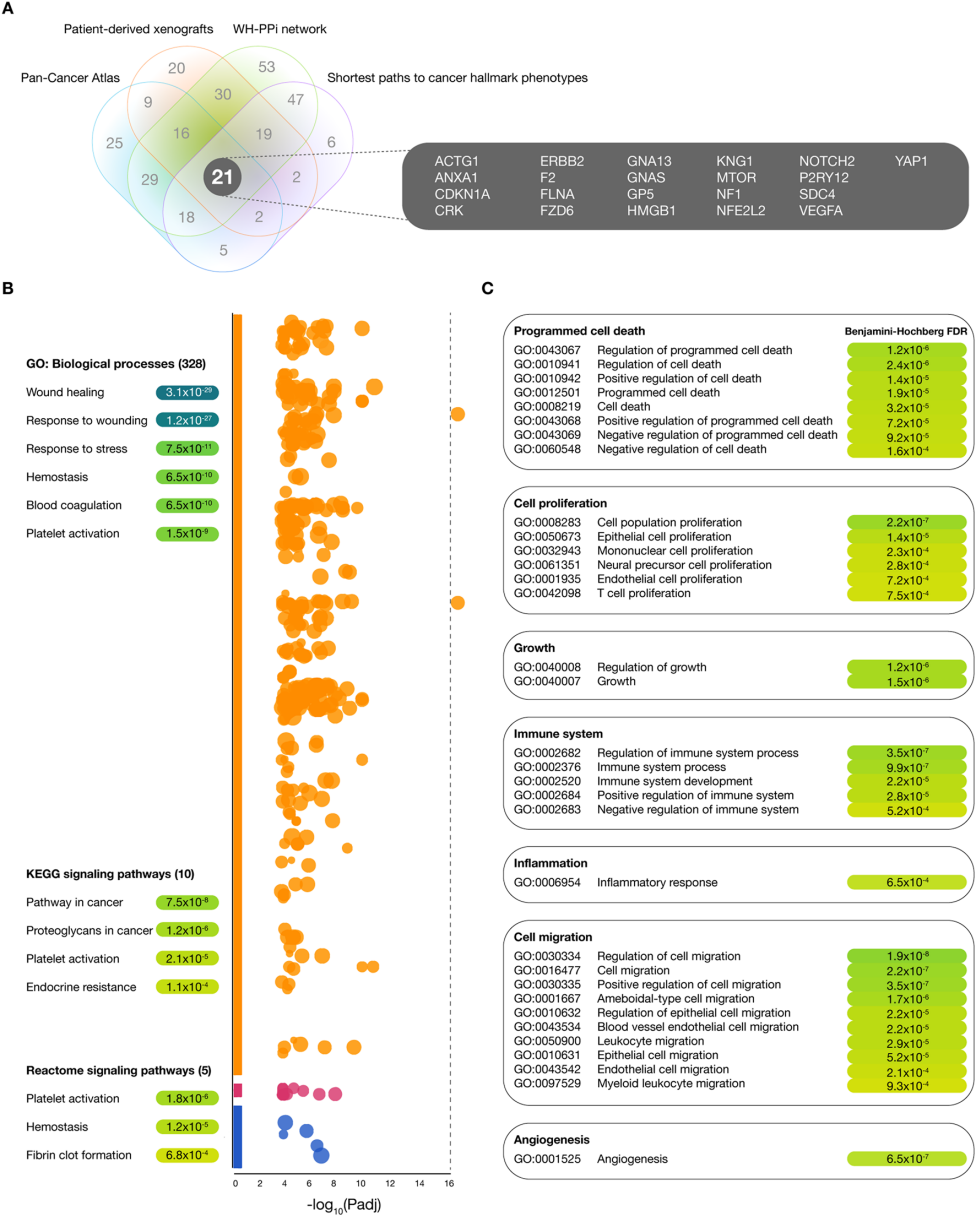
Integration of multi-omics approaches and functional enrichment analysis. (**A**) Venn diagram shows 21 wound healing proteins significantly expressed in the Pan-Cancer Atlas, the patient-derived xenografts, the wound healing protein–protein interactome network, and the shortest paths to cancer hallmark phenotype approaches. (**B**) Functional enrichment analysis showing a Manhattan plot of the most significant GO: biological processes, KEGG signaling pathways^107^, and Reactome signaling pathways^39^. (**C**) Functional enrichment analysis showing the most significant GO: biological processes related to hallmarks of cancer (programmed cell-death, cell proliferation, cell growth, immune system, inflammation, cell migration, and angiogenesis). Significant annotations were calculated through the Benjamini–Hochberg FDR *q* < 0.001. Lastly, the functional enrichment analysis was visualized by using the g:Profiler software version e101_eg48_p14_baf17f0 (https://biit.cs.ut.ee/gprofiler/gost)^37,38^.

Figure 6A shows a Venn diagram integrating the multi-omics approaches (PCA, PDXs, WH-PPi network, and the shortest paths to cancer hallmark phenotypes), and obtaining the 21 most relevant WH proteins in our study (Supplementary Table 18). Subsequently, we performed a functional enrichment analysis of these 21 WH proteins, obtaining 328 GO: biological processes, 10 KEGG signaling pathways, and 5 Reactome signaling pathways as shown in the Manhattan plot of Fig. 6B. The most significant GO: biological processes with Benjamini–Hochberg FDR *q* < 0.001 were wound healing (GO:0042060, 3.1 × 10^–29^), response to wound healing (GO:0009611, 1.2 × 10^–27^), response to stress (GO:0006950, 7.5 × 10^–11^), hemostasis (GO:0007599, 6.5 × 10^–10^), blood coagulation (GO:0007596, 6.5 × 10^–10^), platelet activation (GO:0030168, 1.5 × 10^–9^), among others. The most significant KEGG signaling pathways were pathways in cancer (KEGG:05200, 7.5 × 10^–8^), proteoglycans in cancer (KEGG:05205, 1.2 × 10^–6^), platelet activation (KEGG:04611, 2.1 × 10^–5^), and endocrine resistance (KEGG:01,522, 1.1 × 10^–4^). Lastly, the most significant Reactome signaling pathways were platelet activation (REAC:R-HSA-76002, 1.8 × 10^–6^), hemostasis (REAC:R-HSA-109582, 1.2 × 10^–5^), and intrinsic pathway of fibrin clot formation (REAC:R-HSA-140837, 6.8 × 10^–4^) (Fig. 6B and Supplementary Table 19).

### Hallmarks of cancer and wound healing

**Figure 7:**
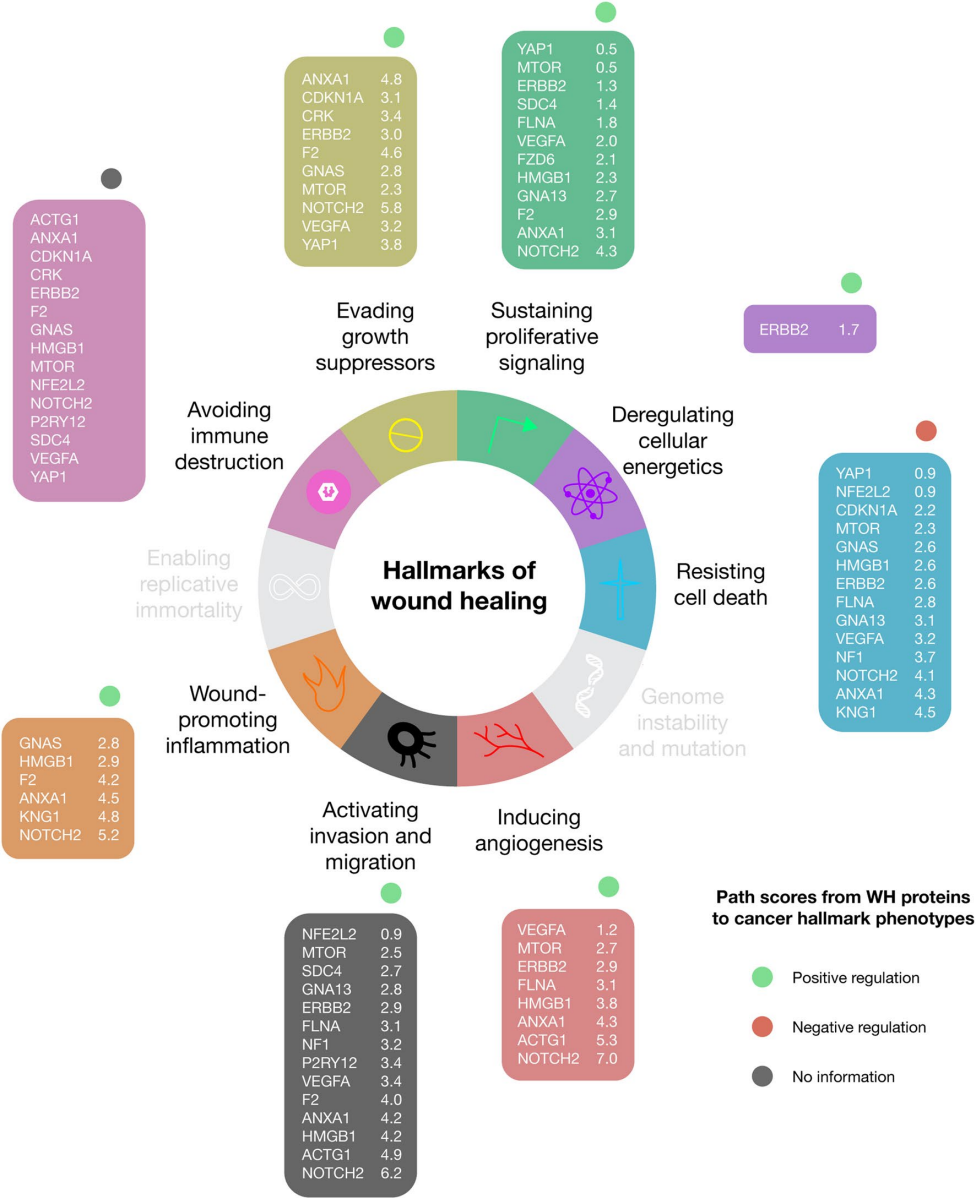
Key proteins involved in both hallmarks of cancer and hallmarks of wound healing. Additionally, each protein set was ranked according to the shortest paths from wound healing proteins to cancer hallmark phenotypes. Path scores of positive regulation were considered for evading growth suppressors, sustaining proliferative signaling, deregulating cellular energetics, inducing angiogenesis, activating invasion and migration, and wound-promoting inflammation; path scores of negative regulation were considered for resisting cell death; and avoiding immune destruction does not show path score information.

The hallmarks of cancer constitute an organizing principle for rationalizing the complexities of neoplastic disease. Nowadays, there are ten biological capabilities acquired during the multistep development of human tumors: (1) sustaining proliferative signaling, (2) evading growth suppressors, (3) resisting cell death, (4) enabling replicative immortality, (5) inducing angiogenesis, (6) activating invasion and metastasis, (7) genome instability, (8) inflammation, (9) reprogramming of energy metabolism, and (10) evading immune destruction^2^. On the one hand, six WH proteins of our study were already catalogued as key proteins in six hallmarks of cancer features according to the COSMIC and CGC databases^19^. NFE2L2, MTOR, and ERBB2 promotes change of cellular energetics; NOTCH2, MTOR, GNAS, and ERBB2 promotes proliferative signaling; MTOR promotes angiogenesis and NF1 suppresses angiogenesis; NOTCH2, NF1, and GNAS promotes suppression of growth; MTOR promotes invasion and metastasis and NF1 suppresses invasion and metastasis; and, NOTCH2, NFE2L2, and MTOR promotes escaping programmed cell death and NF1 suppresses escaping programmed cell death (Fig. 7 and Supplementary Table 20). On the other hand, 21 of our most relevant WH proteins (ACTG1, ANXA1, CDKN1A, CRK, ERBB2, F2, FLNA, FZD6, GNA13, GNAS, GP5, HMGB1, KNG1, MTOR, NF1, NFE2L2, NOTCH2, P2RY12, SDC4, VEGFA, and YAP1) had shortest paths of positive regulation to the cancer hallmark phenotypes whose path scores are detailed in Fig. 7 and Supplementary Table 17.

Additionally, MacCarthy-Morrogh & Martin highlighted how tissue repair and cancer share cellular and molecular processes that are regulated in a wound but misregulated in cancer^4^. They proposed eight prospective hallmarks that might apply to both cancer and wound healing: (1) avoiding immune destruction, (2) wound-promoting inflammation, (3) activating invasion and migration, (4) inducing angiogenesis, (5) resisting cell death, (6) sustaining proliferative signaling, (7) evading growth suppressors, and 8) deregulating cellular energetics.

Subsequently, the functional enrichment analysis performed on our 21 most relevant WH proteins addressed significant annotations (Benjamini–Hochberg FDR *q* < 0.001) between 20 WH proteins and hallmarks shared by cancer and wound healing. ANXA1, CDKN1A, FLNA, GNA13, HMGB1, KNG1, MTOR, NF1, NFE2L2, NOTCH2, YAP1, and VEGFA were involved in 8 significant annotations related to cell death (GO:0043067, 1.2 × 10^–6^; GO:0010941, 2.4 × 10^–6^; GO:0010942, 1.4 × 10^–5^; GO:0012501, 1.9 × 10^–5^; GO:0008219, 3.2 × 10^–5^; GO:0043068, 7.2 × 10^–5^; GO:0043069 9.2 × 10^–5^; and GO:0060548, 1.6 × 10^–4^); ANXA1, CDKN1A, ERBB2, F2, FLNA, FZD6, GNA13, HMGB1, MTOR, NF1, NOTCH2, SDC4, YAP1, and VEGFA were involved in 6 significant annotations related to cell proliferation (GO:0008283, 2.2 × 10^–7^; GO:0050673, 1.4 × 10^–5^; GO:0032943, 2.3 × 10^–4^; GO:0061351, 2.8 × 10^–4^; GO:0001935, 7.2 × 10^–4^; and GO:0042098, 7.5 × 10^–4^); ANXA1, CDKN1A, CRK, ERBB2, F2, GNAS, MTOR, NOTCH2, YAP1, and VEGFA were involved in 2 significant annotations related to growth (GO:0040008, 1.2 × 10^–6^; and GO:0040007, 1.5 × 10^–6^); ACTG1, ANXA1, CDKN1A, CRK, ERBB2, F2, GNAS, HMGB1, MTOR, NFE2L2, NOTCH2, P2RY12, SDC4, YAP1, and VEGFA were involved in 5 significant annotations related to the immune system (GO:0002682, 3.5 × 10^–7^; GO:0002376, 9.9 × 10^–7^; GO:0002520, 2.2 × 10^–5^; GO:0002684, 2.8 × 10^–5^; and GO:0002683, 5.2 × 10^–4^); ANXA1, F2, GNAS, HMGB1, KNG1, NFE2L2, and NOTCH2 were involved in 1 significant annotation related to the inflammatory response (GO:0006954, 6.5 × 10^–4^); ACTG1, ANXA1, CRK, F2, FLNA, GNA13, HMGB1, MTOR, NF1, NF2L2, P2RY12, SDC4, and VEGFA were involved in 10 significant annotations related to cell migration (GO:0030334, 1.9 × 10^–8^; GO:0016477, 2.2 × 10^–7^; GO:0030335, 3.5 × 10^–7^; GO:0001667, 1.7 × 10^–6^; GO:0010632, 2.2 × 10^–5^; GO:0043534, 2.2 × 10^–5^; GO:0010631, 5.2 × 10^–5^; GO:0043542, 2.1 × 10^–4^; and GO:0097529, 9.3 × 10^–4^); and ACTG1, ANXA1, ERBB2, FLNA, GNA13, HMGB1, MTOR, NF1, NF32L2, P2RY12, SDC4, and VEGFA were involved in 1 significant annotation related to angiogenesis (GO:0001525, 6.5 × 10^–7^) (Figs. 6C and Supplementary Tables 19 and 21). Lastly, Fig. 7 details the key proteins in the signaling crossroad between cancer hallmark phenotypes and wound healing.

## Discussion

Proteins involved in cancer hallmarks may play a crucial role in WH processes and vice versa*,* mediating tumor progression or tissue reconstitution. The identification of these proteins and understanding how they work in cancer or WH, could lead to new therapeutic approaches. We identify a set of 21 proteins that control processes related to the cancer hallmarks and WH. Interestingly, TCGA Pan-Cancer types with the highest means of alteration in WH proteins are carcinomas, solid tumors of epithelial origin. Non-solid tumors, such as leukemia, do not show a high alteration mean for WH proteins. Evidence shows that leukaemias and solid tumors could have a common hierarchical organization in terms of carcinogenesis, being originated from a cancer stem cell with sustained self-renewing capacities, which gives rise to other cells with a distinct phenotype, generating cell diversity^41^. Even if a cancer stem cell or a cell-of origin of cancer could exist among solid and non solid-tumors, cells could diverge from these progenitors and develop characteristics associated with their tissue of origin^42–46^. In this sense, the identified non-solid tumors could maintain or develop the use of WH proteins as support for their progression, modifying the tumor microenvironment, perturbing normal WH processes^47–49^. Leukemic cells have completely different ways of interacting with their environment thus, not using the WH-healing proteins found in solid-tumors^50^.

Among the alterations with the highest frequency mean, we found that mRNA high and CNV amplification were the most represented in the 347 WH genes/proteins analysed through the TCGA Pan-Cancer Atlas. Cancer is characterized by many genomic variations where a close correlation between CNVs and differential gene expression have a qualitative relationship with its downstream effect, especially for oncogenes and tumor suppressor genes^51^. The trend observed by Shao et al., through the analysis of the copy number and mRNA expression data of Broad-Novartis Cancer Cell Line Encyclopedia (CCLE), NCI-60 and TCGA, showed similarities with our study regarding the mRNA high and CNV amplification frequency in solid tumors. Similarly, CNVs change the expression levels of genes located in the concerned genomic region^52^. However, a different set of genes were identified among studies due to the focus of our work in WH genes associated in cancer. Understanding the relationship between WH genes in cancer, their CNV (in germ cells), CNA (in somatic cells) and gene expression characteristics could help in the development of better diagnostic tools and therapeutics^52,53^.

Recently, processes involved in cell survival and proliferation that might apply to both cancer and WH have been proposed as converging hallmarks, grouping common pathways of regulation and activation. In particular, eight of the ten recognized cancer hallmarks proposed by Hanahan and Weinberg have been related with WH processes^4^. We used these eight recently proposed wound healing hallmarks to classify groups of genes/proteins that participate in WH (Fig. 7). “Avoiding immune destruction” (15 proteins), “resisting cell death” (14 proteins), and “activating invasion and migration” (14 proteins) were the hallmarks with the most identified proteins. In wound healing, avoiding immune destruction and resisting cell death, could be linked to a stress resilient mechanism, helping cancer cells to be detected and killed by immune detection and the collateral damage of reactive oxygen species released by immune cells^4,54^. The large number of proteins grouped on avoiding immune cells could be associated with a reduction in the pro-inflammatory activity in the tumor. This could be related to the regulation of the inflammatory phase in wound healing, whereas in cancer it is more involved in the down-regulation of immune recognition and increased immune escape^2^. On the other hand, only one protein, ERBB2, was associated with the proposed WH hallmark of “deregulating cellular energetics,” interestingly common to all hallmarks of wound healing except “wound promoting inflammation”. However, it is well known that normal proliferating cells such as the ones involved in wound healing exhibit a metabolic switch from oxidative phosphorylation to an aerobic glycolytic pathway as seen in the ‘Warburg effect’ in cancer cells^55–57^. Previous transcriptomic analysis revealed metabolic heterogeneity in wounded mouse skin with an increased expression of proteins associated with glycolysis and a reduction of transcripts linked to oxidative phosphorylation^58^. In addition, some of the identified genes in this work code for proteins such as mTOR which has an important role in mediating changes in cellular metabolism and promoting glycolysis^57,59^. Even though WH is a natural physiological process for tissue repair, and cancer involves an abnormal state, the common mTOR expression is proposed to facilitate the uptake of nutrients demanded for cell growth and proliferation in both processes^55^.

We identified 21 WH proteins in the majority of cancer/WH hallmarks, we found among the most represented: ANXA1 and NOTCH2 (found in 7 of 8 hallmarks, excluding “deregulating cellular energetics”), ERBB2 (found in 7 of 8 hallmarks, excluding “wound promoting inflammation”), mTOR and VEGFA (found in 6 of 8 hallmarks, excluding “deregulating cellular energetics” and “wound promoting inflammation”), HMGB1 (found in 6 of 8 hallmark, excluding “evading growth suppressors” and “deregulating cellular energetics”), FLNA (found in 4 of 8 hallmarks, excluding “inflammation”, “evading growth suppressor”, “avoiding immune destruction” and “deregulating cellular energetics”), and YAP1 (found in 4 of 8 hallmarks, excluding “deregulating cellular energetics, “inflammation”, “angiogenesis” and “activating invasion and migration”. The identification of these WH proteins involved in most of the cancer/WH hallmarks represent a major opportunity to understand how the regulation of the WH would be applied to stop cancer growth.

These proteins common to most of the cancer/WH hallmarks are associated with proliferation and migration processes both key for WH and cancer progression. The Notch signaling pathway is evolutionarily conserved among multicellular organisms regulating stem cell maintenance, cell proliferation, differentiation, and apoptosis^59–61^. These key effects are important for the control of cancer and proper stimulation of the WH process^62^. In mammals, one of the four types of Notch proteins, NOTCH2 promotes angiogenesis with an important role in the carcinogenesis process. Interestingly, it has been observed that NOTCH1 controls NOTCH2, thus acting as a tumor suppressor. NOTCH2 promotes immune activation and inflammations modulating macrophages phenotype^63,64^. The proinflammatory role of NOTCH2 is detrimental for the healing of diabetic wounds, being an interesting target of molecules with regenerative properties^65,66^.

ERBB2 plays a crucial role in cell proliferation, epithelial differentiation and WH^67^. It has been reported that ERBB2 promotes tumor progression especially in skin and breast cancers^68,69^. In wound healing, the ERBB2 activation during the proliferative phase of tissue repair promotes the WH of human airway epithelial cell wounds. The blockage of ERBB2 leads to the failure of the regenerative process. However, blocking ERBB2 could be an important therapeutic target in cancer^67,70,71^.

The activity of mTOR, as mentioned before, is considered a central regulator of cell growth, proliferation, cellular metabolism, survival and homeostasis^72,73^. mTOR plays an important role in the PI3K-AKT signaling pathway involved in WH mechanisms such as growth and proliferation. However, evidence shows that mTOR deregulation is implicated in tumorigenesis and tumor progression. In WH, mTOR improves the wound closure rate, being especially active in epithelial cells. The pharmacological activation of PI3K-AKT-mTOR improves WH, influencing its upstream regulators PTEN and TSC1^74^. The deregulation of mTOR is implicated in progression of cancer and the aging process^75^. This research provides more evidence of the important role of mTOR in WH and cancer being a key target for therapy, whether promoting tissue regeneration or by preventing cancer growth when blocked.

In the tumor microenvironment and during WH, the physical interaction of fibroblasts with collagen regulate many cellular processes through the activity of FLNA. FLNA is an actin filament cross-linking protein that acts in the regulation of cell adhesion^76^. Several actin-binding proteins, including vinculin, α-actinin, paxillin, talin, cortactin, gelsolin, and filamins provide instructive signals that regulate the maintenance of tension in collagen during remodeling. This is linked to the FLNA function which focuses on mediating cell-induced contraction and wound closure^77,78^. On the other hand, FLNA can induce two opposite outcomes in cancer depending on its subcellular localization: (1) promoting cell growth and metastasis when it is present in the cytoplasm and interacting with cell signalling molecules, or (2) inhibiting cell growth and preventing metastasis when it is acting in the nucleus and interacting with transcription factors^79^. In cancer, the inhibition of NF1 contributes to the generation of melanomagenesis by enhancing the activation of PI3K signaling, this inhibition favours angiogenesis, escaping apoptosis, migration, and cancer cell invasion^80–83^.

The YAP1 protein, a precursor of the activity of the YAP/TAZ signaling pathway, is essential for skin homeostasis and WH^84,85^. YAP1 has been associated with processes such as proliferation and the recruitment of M2 macrophages, myeloid-derived suppressor cells (MDSC), and regulatory T cells to suppress host effector T cells in the tumor microenvironment^86^. YAP1 activity in tumors results in cancer progression and drug resistance^86^. YAP1 contributes to cancer invasion and migration by promoting SNAI2 transcription through the transcription cofactor TEAD, in vivo and in vitro assays. YAP1 expression promotes cell proliferation, migration, and invasion, while silencing YAP1 significantly inhibits cell migration, invasion, and growth^84^. Given that YAP1 is hyperactive in many human cancers, it suggests that therapeutic targeting of YAP1 could regulate key processes in the tumor, thereby disrupting its survival mechanisms^87^.

During the WH process, the inhibition of thrombospondin 1 (TSP-1) and 2 (TSP-2) by HRG promotes angiogenesis facilitaning wound closure^88^. Similarly, ANXA1 promotes the development of blood vessels and metastasis with a deleterious effect in cancer, however positive effects during the WH process^89,90^.

Among the least represented proteins, we found NFE2L2 and CDKN1A. NFE2L2 is activated after tissue damage and promotes wound repair protecting cells for the damaging effects of ROS production during inflammation^91^. Insufficient NFE2L2 expression impairs WH in a process associated with severe tissue damage and uncontrolled inflammation^92^. Inhibition of NFE2L2 in keratinocytes favors tumor development. In contrast, when NFE2L2 is functional, it plays an important role in reducing cellular stress, preventing DNA damage and cancerous mutations^93^. NFE2L2 expresses a bZIP protein with cytoprotective effects^94,95^, regulating genes involved in redox homeostasis^56,96,97^, this functioning in the stress defense in mammalian cells^98^. However, when NFE2L2 is overexpressed in cancer cells, it promotes their drug and radiotherapy resistance^99^. The *CDKN1A* gene was initially considered as a potential tumor suppressor. However, studies have reported that this gene could act as an oncogene due to its anti-apoptotic activities^100,101^. It is involved in p53-mediated inhibition of cell proliferation in response to DNA damage leading to cell cycle arrest at the G1/S checkpoint^102,103^. By overexpressing CDKN1A, wound fibroblasts enhance age-related healing, creating potential clinical avenues to promote wound healing in the elderly population^104^. Inhibition of this protein can act to enhance the regenerative response in several ways, altering DNA damage and checkpoint responses, leading to increased proliferation. It can reduce TGF-β signaling, which decreases scar formation and would alter differentiation patterns^105^.

This work identified 21 proteins in which eight have shown to be highly associated between WH and the cancer hallmarks. These proteins could represent critical nodes in the network of a controlled WH process or a deregulated interaction leading to tumor progression. Interestingly, the identified proteins were highly expressed only in solid tumors, as cancer cells must use them to modify the tissue microenvironment and induce immune cell regulation. This protein set could be specially needed to reorganize the wounded tissue and lead to regeneration. However, in the case of cancer, these proteins induce immune escape and survival. Putting in evidence these common proteins between WH and cancer brought light to those principally involved in sustained proliferation, invasion, angiogenesis, and others connected and overlapped between health and disease. Further interdisciplinary studies in vivo or in patients will support the in silico role of the identified and overlapped proteins. Interdisciplinarity is important as it brings together specialists in bioinformatics, health care professionals and technical experts leading to the development of better screening methods for early diagnosis and therapies^106^. These studies would allow therapeutic targeting and the development of specific pharmacological compounds that would stop cancer progression without affecting the healing process.

## Supplementary Information

Supplementary Information.

## Data Availability

The datasets generated for this study are included in this published article (and its Supplementary Information files).
